# Electronic Features of Cotton Fabric e-Textiles Prepared with Aqueous Carbon Nanofiber Inks

**DOI:** 10.1021/acsaenm.2c00023

**Published:** 2022-09-15

**Authors:** Antonio. J. Paleo, Beate Krause, Maria Fátima Cerqueira, Enrique Muñoz, Petra Pötschke, Ana Maria Rocha

**Affiliations:** † 2C2T-Centre for Textile Science and Technology, University of Minho, Campus de Azurém, 4800-058 Guimarães, Portugal; ‡ Leibniz-Institut für Polymerforschung Dresden e.V. (IPF), Hohe Str. 6, 01069 Dresden, Germany; § INL-International Iberian Nanotechnology Laboratory, Av. Mestre. Jose Veiga, 4715-330 Braga, Portugal; ∥ CFUM − Center of Physics of the University of Minho, Campus de Gualtar, 4710-057 Braga, Portugal; ⊥ Facultad de Física, Pontificia Universidad Católica de Chile, Santiago 7820436, Chile

**Keywords:** carbon nanofibers, cotton fabrics, aqueous conductive inks, surfactant, e-textiles, Seebeck coefficient, variable range hopping

## Abstract

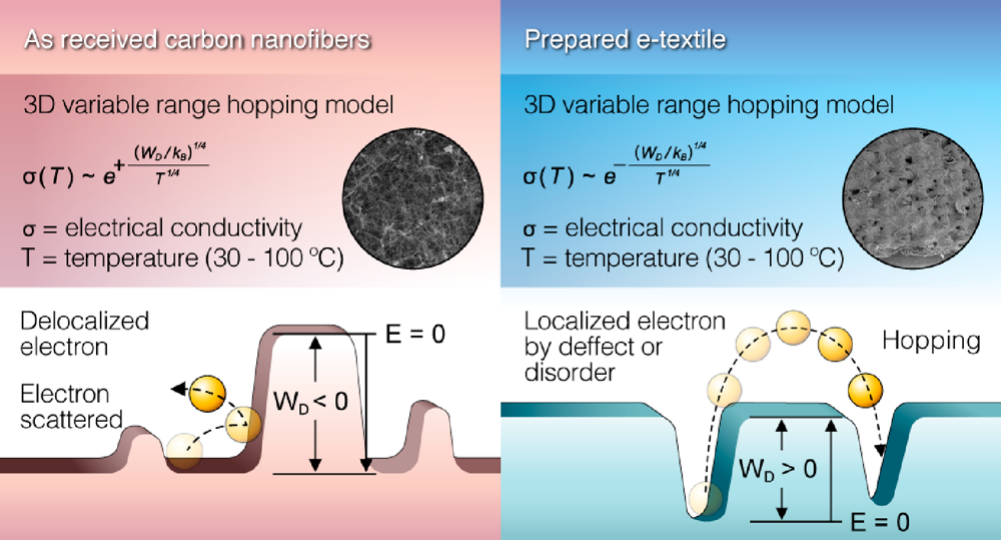

Cotton woven fabrics functionalized with aqueous inks made with carbon nanofibers (CNFs) and anionic surfactant are prepared via dip-coating followed by heat treatment, and their electronic properties are discussed. The e-textiles prepared with the inks made with the highest amount of CNFs (6.4 mg mL^–1^) show electrical conductivities (σ) of ∼35 S m^–1^ and a negative Seebeck (*S*) of −6 μV K^–1^ at 30 °C, which means that their majority carriers are electrons. The σ(*T*) of the e-textiles from 30 to 100 °C shows a negative temperature effect, interpreted as a thermally activated hopping mechanism across a random network of potential wells by means of the 3D variable range hopping (VRH) model. Likewise, their *S*(*T*) from 30 to 100 °C shows a negative temperature effect, conveniently depicted by the same model proposed for describing the negative Seebeck of doped multiwall carbon nanotube mats. From this model, it is deduced that the cause of the negative Seebeck in the e-textiles may arise from the contribution of the impurities found in the as-received CNFs, which cause sharply varying and localized states at approximately 0.085 eV above their Fermi energy level (*E*_F_). Moreover, the possibility of a slight n-doping from the cellulose fibers of the fabrics and the residuals of the anionic surfactant onto the most external CNF graphitic shells present in the e-textiles is also discussed with the help of the σ(*T*) and *S*(*T*) analysis.

## 1 Introduction

Electronic textiles (e-textiles) impart conductive functionality in conventional textiles without altering the intrinsic textile characteristics of strength, flexibility, durability, comfort, etc.^1^ The production of e-textiles requires, first, the use of conductive materials and, second, the use of scalable methods to apply them in textiles. Regarding the first condition, there is a wide variety of materials such as metals, conductive carbon allotropes, and conjugated polymers that can be used in the production of e-textiles.^2^ Among them, carbon-based materials such as carbon black (CB), carbon fibers (CFs), carbon nanofibers (CNFs), carbon nanotubes (CNTs), graphite, graphene, and its derivatives are extensively used for the manufacturing of e-textiles because the availability of their raw materials and increasingly low-cost processes.^3^ Usually, these sorts of materials are solids, which makes their application into textiles difficult. Therefore, one simple option is to suspend them in a fluid to form a conductive ink that can be applied in textiles.^4^ Essentially, there are two approaches to produce e-textiles. The bottom-up approach relies on producing functional fibers or yarns by fiber-spinning technologies that are transformed into e-textiles with methods such as weaving, knitting, embroidery, and braiding techniques.^5^ The top-down approach, on the other hand, consists of utilizing a final textile product that it is transformed into an e-textile through various strategies such as screen-printing, inkjet-printing, spray-coating, and dip-coating, which are the most common methods.^6^

Though the range of applications where e-textiles can be utilized is huge, they can be grouped into three main categories: e-textiles for sensors and e-textiles for electricity generation and storage.^7^ More importantly, in order to achieve significant advances in all these applications, it is necessary to establish direct relationships between the electronic properties such as the majority carrier type and electrical conductivity (σ) of the functional conductive materials and their resulting e-textiles. In this respect, the Seebeck coefficient (*S*), which reflects the voltage produced in a semiconductor when subjected to a thermal gradient, allows to know the majority carrier type present in the semiconductor.^8^ It should be reminded that the majority carrier type of a semiconductor defines its ultimate use in a large variety of devices from thermoelectric modules to solar cells.^9^ Thus, n-type semiconductors have a negative *S* (majority of electrons), while p-type semiconductors hold positive *S* values (majority of holes).^10^

It is in this context that aqueous inks made by dispersing different contents of carbon nanofibers with sodium dodecylbenzenesulfonate (SDBS) are used in this work to prepare coated cotton woven textile fabrics with electrical functionality. In the case of the anionic surfactant SDBS used in this study, the hydrophobic tail of the surfactant molecule adsorbs on the surface of CNF bundles, while the hydrophilic head associates with water. By this mechanism of hydrophobic and hydrophilic interactions, the bundles or agglomerates are ideally separated into individual CNFs and are kept in homogeneous and stable suspension.^11^ Then, a comprehensive analysis is done through the comparison between electronic properties (σ and *S*) of the as-received CNFs in powder form used and the e-textiles prepared with the aqueous CNF inks. The e-textiles showed lower σ than the as-received CNFs. In contrast, quite unexpectedly, the e-textiles showed even more negative *S* (higher absolute values). Thus, it is confirmed that the CNFs can transfer their intrinsic n-type character (majority of electrons) to the e-textiles.^12^ Notably, the σ(*T*) (σ as a function of temperature between 30 and 100 °C) of the e-textiles cannot be explained only in terms of the σ(*T*) found in CNFs. This is reflected in the fact that, while the σ(*T*) of the CNFs present positive temperature effect or dσ/d*T* < 0, the e-textiles show a negative temperature coefficient effect (dσ/dT > 0). Moreover, through the modeling of *S*(*T*) (*S* as a function of temperature between 30 and 100 °C) of the CNF powder and the e-textiles, the origin of the n-type character of this type of CNFs, and the reason behind the higher values of *S* found in the e-textiles, could be deduced. All these results are properly detailed in the subsequent sections to help to correlate the electronic properties of the e-textiles prepared with a simple methodology with device functionality, a key practice for optimizing optoelectronic applications such as solar cells, all type of sensors (physical and chemical), and thermoelectric devices that may utilize e-textiles as building blocks.

## 2 Experimental Section

### 2.1 Materials and Processing

**Figure 1:**
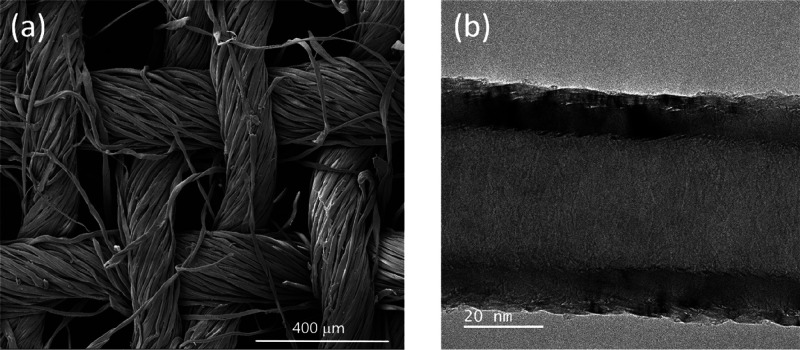
Morphology of cotton woven fabric and carbon nanofiber: (a) SEM of CWF and (b) TEM of single CNF.

In this study, 100% cotton woven fabric (CWF) provided by Somelos Tecidos (Portugal) is used as a support material as provided by the manufacturer. Its physical properties and constructional parameters are listed in Table 1, while its morphology can be seen in Figure 1a. In short, the CWF consists of warp and weft yarns with squared voids of around 250 × 250 μm^2^ between them. Carbon nanofibers produced by chemical vapor deposition (CVD), Pyrograf-III PR 24 LHT XT, (ASI, Cedarville, OH), were selected to provide the cotton fabric with electrical functionality. Details about Pyrograf-III CNFs can be found in previous reports.^13,14^ Briefly, the CNFs are grown at 1100 °C with a thermal post-treatment in an inert atmosphere at 1500 °C, which morphologically results in a dual wall structure surrounding the hollow tubular core as shown in Figure 1b. The CNFs have bulk densities between 0.016 and 0.048 g cm^–3^ and a range of lengths of 30–100 μm. All the other materials used in this work were purchased from Sigma-Aldrich, and they were used without further purification.

**Table 1:** Constructional Parameters and Physical Properties of the Cotton Woven Fabrics Used in This Study

fabric parameters	CWF
weave pattern	1/1 plain
linear density (tex)	14.9 × 20.2
warp × weft yarns (cm^–1^)	35.0 × 14.0
fabric mass (g m^2^)	93.35
fabric thickness at 18 Pa (mm)	0.26
fabric density (g cm^–3^)	0.359
fabric porosity (%)^a^	76.7

a Porosity (%) = 1– [fabric density (g cm^–3^)/ fiber density (for cotton, 1.54 g cm^–3^)] × 100.

CNFs concentrations of 1.6, 3.2, and 6.4 mg·mL^–1^ were added to 5 mg·mL^–1^ SDBS dissolved in distilled water (DI). The solutions were dispersed through tip sonication (ultrasonic homogenizer CY-500; 60% power, 5 min) to obtain the conductive inks utilized for the dip-coating process. Pristine fabrics (2 × 2 cm^2^) were dipped for 5 min and then dried at 80 °C for 10 min. This process was repeated five times. After, the samples were washed by dipping during 10 min in DI, followed by drying at 80 °C for 10 min. This washing step was repeated four times. Finally, a final dipping in ethanol and drying at 80 °C during 10 min was made to ensure as much as possible the elimination of SDBS. At the end, three different types of dip-coated cotton fabrics hereafter referred as e-textiles CWF@1.6 CNF, CWF@3.2 CNF, and CWF@6.4 CNF were produced.

### 2.2 Morphological and Structural Analysis

The CNFs were imaged with a JEOL JEM-2100 electron microscope operating a LaB6 electron gun at 80 kV and acquired with an “OneView” 4k × 4k CCD camera at minimal under-focus to get the surface layers of the CNFs visible. The morphological analysis of CWF and e-textiles were carried out in an ultrahigh resolution field emission gun scanning electron microscope (FEG-SEM), NOVA 200 Nano SEM, FEI Company. Raman spectroscopy measurements were carried out on an ALPHA300 R confocal Raman microscope (WITec) using a 532 nm laser for excitation in back scattering geometry. The laser beam with *P* = 0.5 mW was focused on the sample by a × 50 lens (Zeiss), and the spectra were collected with 600 g/mm grating using five acquisitions with a 2 s acquisition time. The X-ray photoelectron spectroscopy (XPS) measurements were performed in an ultrahigh vacuum (UHV) system ESCALAB250Xi (Thermo Fisher Scientific). The base pressure in the system was below 5 × 10^–10^ mbar. XPS spectra were acquired with a hemispherical analyzer and a monochromated X-ray source (Al K_α_ radiation, *h*ν = 1486.6 eV) operated at 15 keV and power 200 W. The XPS spectra were recorded with pass energies of 20 eV, energy steps of 0.1 and 200 eV, and an energy step of 1 eV for high resolution and survey spectra, respectively. The spectrometer was calibrated by setting the Au 4f_7/2_ level to 84.0 eV measured on a gold foil and Ag 2p_3/2_ 932.6 eV on a silver foil. The XPS spectra were peak-fitted using Avantage data processing software. The Shirley-type background subtraction was used for peak fitting, and the quantification was done by using the elemental sensitivity factors provided by the Avantage library.

### 2.3 Thermoelectric Analysis

The Seebeck coefficient and volume resistivity of the e-textiles and CNF powder were determined using the self-constructed equipment TEG at Leibniz-IPF.^15^ Samples with a size of ca. 6 mm width and 15 mm length cut from the dipped fabrics using a scissors were inserted between the copper electrodes with a measurement distance of about 10 mm. The measurements of Seebeck coefficient and volume resistivity were performed on the same strips at the mean temperatures of 303 K (30 °C), 338 K (65 °C), and 373 K (100 °C) using a Keithley multimeter DMM2001 (Keithley Instruments, Cleveland, OH). The volume resistivity was measured at the different mean temperatures using a four-wire technique. The given values represent the arithmetic mean values of 10 measurements. The Seebeck coefficient was measured by applying temperature differences between the two copper electrodes of up to ±8 K (eight steps of 2 K each around the mean temperature). The Seebeck coefficient was calculated as the average of 10 thermovoltage measurements. For the thermoelectric analysis of the CNF powder, an insert consisting of a PVDF tube (inner diameter 3.8 mm, length 16 mm) closed with copper plugs filled with the CNF powder was used.^15^ This procedure was performed five times, and the mean values and standard deviation were calculated. The figure of merit at room temperature of all samples was estimated using a value of thermal conductivity of 0.43 W m^–1^ K^–1^, which was obtained from a previous investigation based on anisotropic paper-like mats of 0.5 vol % of Pyrograf-III CNF.^16^

## 3 Results

### 3.1 Morphological Analysis

The total diameter of 25 individual CNFs was measured and averaged from TEM analysis. CNFs showed total average diameters of around 80 nm (Figure 1b). The inner layer shows a very well organized structure consisting of parallel graphene sheets with angles between 10° and 20° with respect to the hollow core. In contrast, a lower number of graphene sheets, practically parallel to the hollow core, is observed in the outer layer, which causes its size (around 4 nm) to be lower than that of the inner layer (around 10 nm).

**Figure 2:**
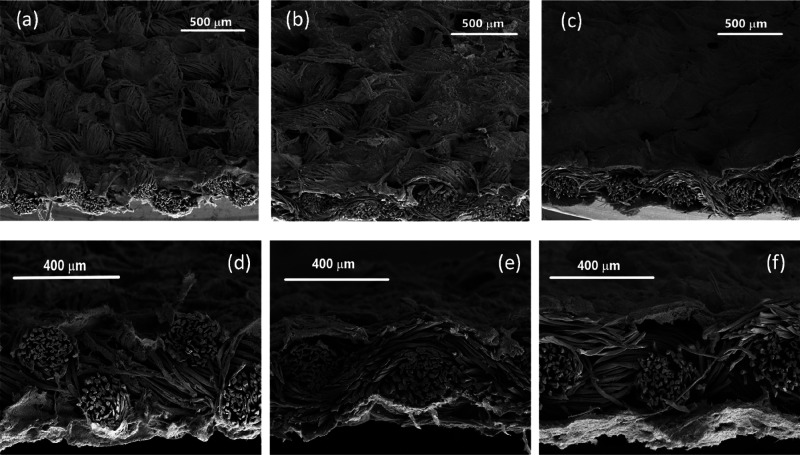
SEM micrographs of e-textiles and their cross sections: (a and d) CWF@1.6 CNF, (b and e) CWF@3.2 CNF reprinted with permission from ref (12), and (c and f) CWF@6.4 CNF.

The SEM micrographs of the e-textiles are shown in Figure 2. The starting cotton fabric structure is clearly noticed on the surface of CWF@1.6 CNF (Figure 2a) and CWF@3.2 CNF (Figure 2b), whereas a sort of CNF mat hides completely the CWF surface in the CWF@6.4 CNF sample (Figure 2c). It is observed from the images taken on the cross sections (Figure 2d–f), that the CNFs are placed mainly on the surface. This feature is not positive since it may facilitate the peeling off the coating from the cotton fabrics more easily. It is expected that the conductive ink made with the highest content of CNFs (6.4 mg mL^–1^) should result in the e-textiles with the highest weights. Thereby, from the difference in weight between the starting and the final fabrics, the CWF@3.2 CNF and CWF@6.4 CNF samples showed values of 1.13 ± 0.27 and 1.61 ± 0.41 mg cm^–2^, respectively. As expected, the CWF@CNF 1.6 samples were the lightest with values of 0.61 ± 0.25 mg cm^–2^. These values correspond with total thicknesses of 0.38 ± 0.02 mm for CWF@1.6 CNF and 0.41 ± 0.03 and 0.47 ± 0.04 mm for CWF@3.2 CNF and CWF@6.4 CNF samples, respectively. It has to be mentioned that pristine CWF has a thickness of 0.26 mm, as it is indicated in Table 1. In conclusion, the different content of CNFs used for producing the conductive waterborne inks affects markedly the surface morphology of e-textiles. Moreover, it is deduced that the infiltration of the CNFs into the space existing within the warp and weft yarns is best promoted in the CWF@3.2 CNF samples after taking into consideration the SEM micrographs together with the weights measured before and after the dip-coating.

### 3.2 Structural Analysis

**Figure 3:**
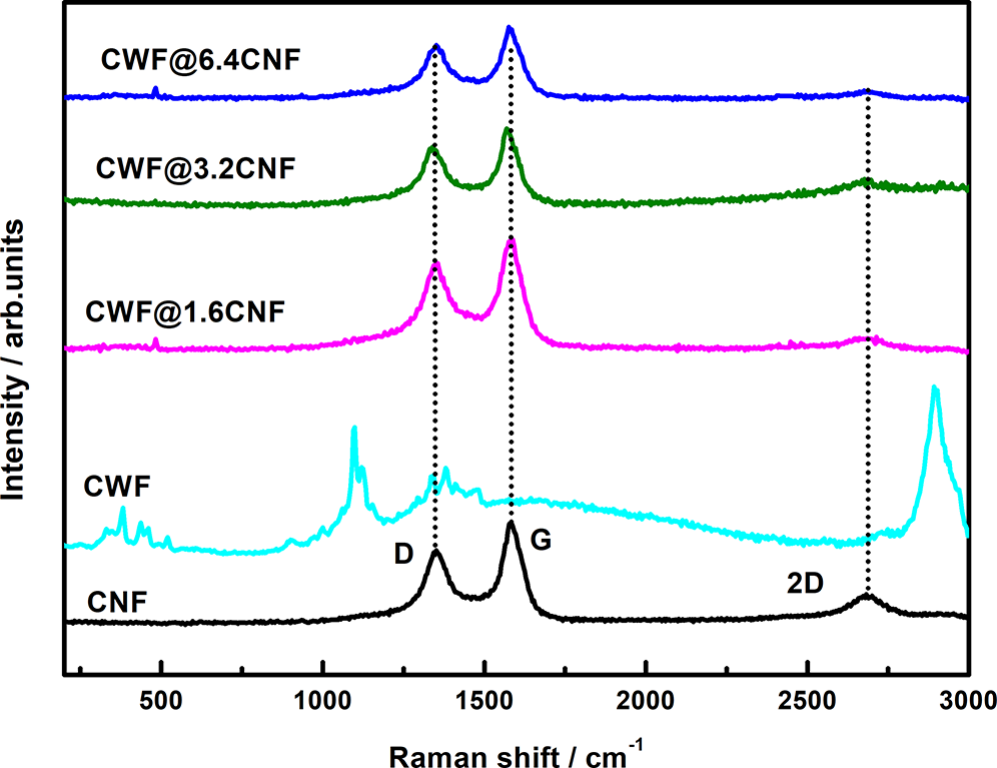
Raman spectra of CNFs, CWF, CWF@1.6 CNF, CWF@3.2 CNF. and CWF@6.4 CNF.

The Raman spectra of CNFs, CWF, and e-textiles CWF@1.6 CNF, CWF@3.2 CNF, and CWF@6.4 CNF are shown in Figure 3. The CNFs present the disorder-induced phonon mode D band at 1350 cm^–1^;^17^ the G-band, characteristic of the graphitic lattice vibration mode and generally used to identify well-ordered CNTs,^18^ at 1580 cm^–1^; and the 2D band, corresponding to a second-order Raman process that involves two phonons close to the zone boundary K point,^19^ at 2700 cm^–1^. The Raman spectra of CWF present the modes observed in cellulose in four ranges: 250–550 cm^–1^ (bending modes involving COC, OCC, and OCO vibrations); 800–1200 cm^–1^ (HCC and HCO bending, COC stretching symmetry, and CO and CC stretching symmetry); 1200–1500 cm^–1^ (HCH, HCC, and HOC wagging, rocking, twisting, and scissoring); and 3000 cm^–1^, corresponding to CH stretching vibrations.^20,21^ Notably, the presence of cellulose is hardly detected in the e-textiles that show essentially the same signature of the CNFs with very slight shifts in the D and G peaks, as it is shown in Table 2. Thus, it assumes that an effective coating is produced. In addition to the peak positions (ω_G_ and ω_D_), Table 2 includes also the full width half-maximum of the D and G modes (fwhm_G,D_), the D and G intensity ratio (*I*_D_/*I*_G_), calculated by fitting the experimental Raman spectra with Lorentzian functions, and the in-plane graphitic domain size (*L*_a_), calculated according to *L*_a_ (nm) = 4.4/(*I*_D_/*I*_G_).^22^ The fwhm_G_ decreases from 85 cm^–1^ in CNFs to 75 and 78 cm^–1^ in the e-textiles. This reduction could be associated to an increase in the order structure of the CNFs coatings induced by the structural geometry of CWF. Finally, very slight variations of *L*_a_ with respect to the pristine CNFs (6.3 nm) were observed for the three CWF@1.6 CNF, CWF@3.2 CNF, and CWF@6.4 CNF samples. In summary, the e-textiles share practically the same Raman spectra observed for the CNFs, without significantly alterations that can be caused by the lower or higher amounts of CNFs present on their surfaces.

**Table 2:** Parameters Obtained from the Fitting of Raman Spectra

sample	ω_G_(cm^–1^)	fwhm_G_ (cm^–1^)	ω_D_ (cm^–1^)	fwhm_D_ (cm^–1^)	*I*_D_/*I*_G_	*L*_a_ (nm)
CNF	1583	85	1352	100	0.70	6.3
CWF@1.6 CNF	1580	75	1347	100	0.75	5.9
CWF@3.2 CNF	1577	75	1345	95	0.77	5.7
CWF@6.4 CNF	1582	78	1350	100	0.74	5.9

**Figure 4:**
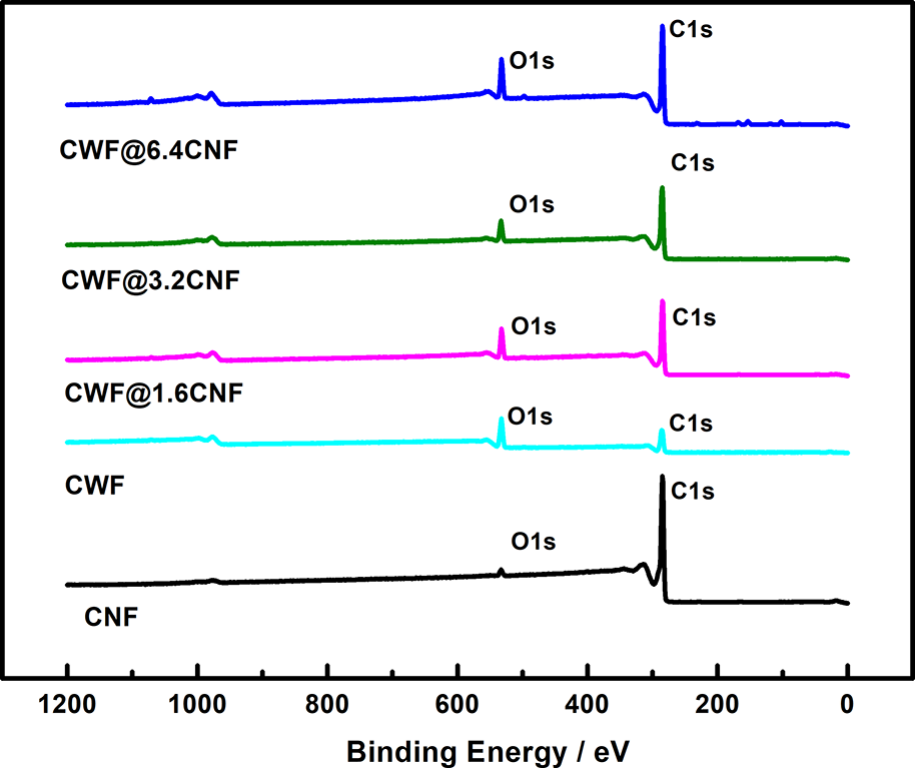
XPS survey spectra of CNFs, CWF, CWF@1.6 CNF, CWF@3.2 CNF, and CWF@6.4 CNF.

The chemical composition of CNFs, CWF, and e-textiles was also analyzed by XPS. All samples contain mainly carbon and oxygen, as it is evidenced by the survey XPS spectra (Figure 4). It is noticeable that traces of sulfur were detected in the as-received CNFs (∼0.1%), as reported in previous studies,^23^ as well as in the CWF@1.6 CNF (0.26%), CWF@3.2 CNF (0.2%), and CWF@6.4 CNF (∼1%) samples, which could also be caused by remaining residues of SDBS used in the formulation of the conductive inks. In addition, the SDBS could also induce the traces of sodium observed in the CWF@1.6 CNF (0.2%) and CWF@6.4 CNF (0.6%) samples. It is significant that Si (3%) and Al (0.8%) were observed in the CWF@6.4 CNF samples, which can be attributed to impurities present in the DI used during the conductive ink production. Table 3 shows the XPS results concerning the C 1s and O 1s contents for carbon sp^2^, adventitious carbon, π–π* satellite, C—O, and C=O species together with the total concentration ratios C/O for all samples. It is noticed that the C/O ratio was similar for samples CWF@1.6 CNF (6.5) and samples CWF@6.4 CNF (6.2), whereas samples CWF@3.2 CNF showed higher C/O ratios of 9.1. This means that, contrary to what could be expected, the XPS did not find the highest amount of C in the samples produced with the highest CNF dispersions (6.4 mg mL^–1^). Interestingly, we can observe that the C—O component associated with the C 1s deconvolution from the pristine substrate CWF (21.3%) decreased significantly in samples CWF@6.4 CNF to 11.4%, which confirms that the signal from the fabric substrate is weaker in these samples. Based on both findings, it can be inferred that the conductive ink used in the CWF@3.2 CNF samples (Figure 2b,e) could facilitate a deeper penetration of CNFs into the whole fabric. Contrarily, CNF agglomerates remain mostly on the surface with the conductive ink used for producing the CWF@6.4 CNF samples, as it is observed in SEM images.

**Table 3:** Summary of the C 1s and O 1s Contents for Carbon sp^2^, Adventitious Carbon, π–π* Satellite, C—O, and C=O Species^a^

		carbon (%)						oxygen (%)		
sample	C/O	C sp^2^	adventitious carbon	C—O	C=O	π–π* satellite	C—O**/**C=O	O—C	O=C	O_total_
CNF	55.8	84.5	–	4.7	–	8.9	–	0.9	0.9	1.8
CWF	2.3	35.3	–	21.3	13.4	–	1.6		30.0	30.0
CWF@CNF1.6	6.5	47.8	13.3	19.1	3.1	–	6.2	0.3	13.1	13.4
CWF@CNF3.2	9.1	49.8	12.2	22.9	5	5.1	4.5	3.3	6.6	9.9
CWF@CNF6.4	6.2	41.1	23.0	11.4	–	2.3	–		13.1	13.1

a Total concentration ratios C/O are also shown. The symbol “–” means that the component was not detected. Empty space means that the component was detected but its content was not calculated by the deconvolution.

**Figure 5:**
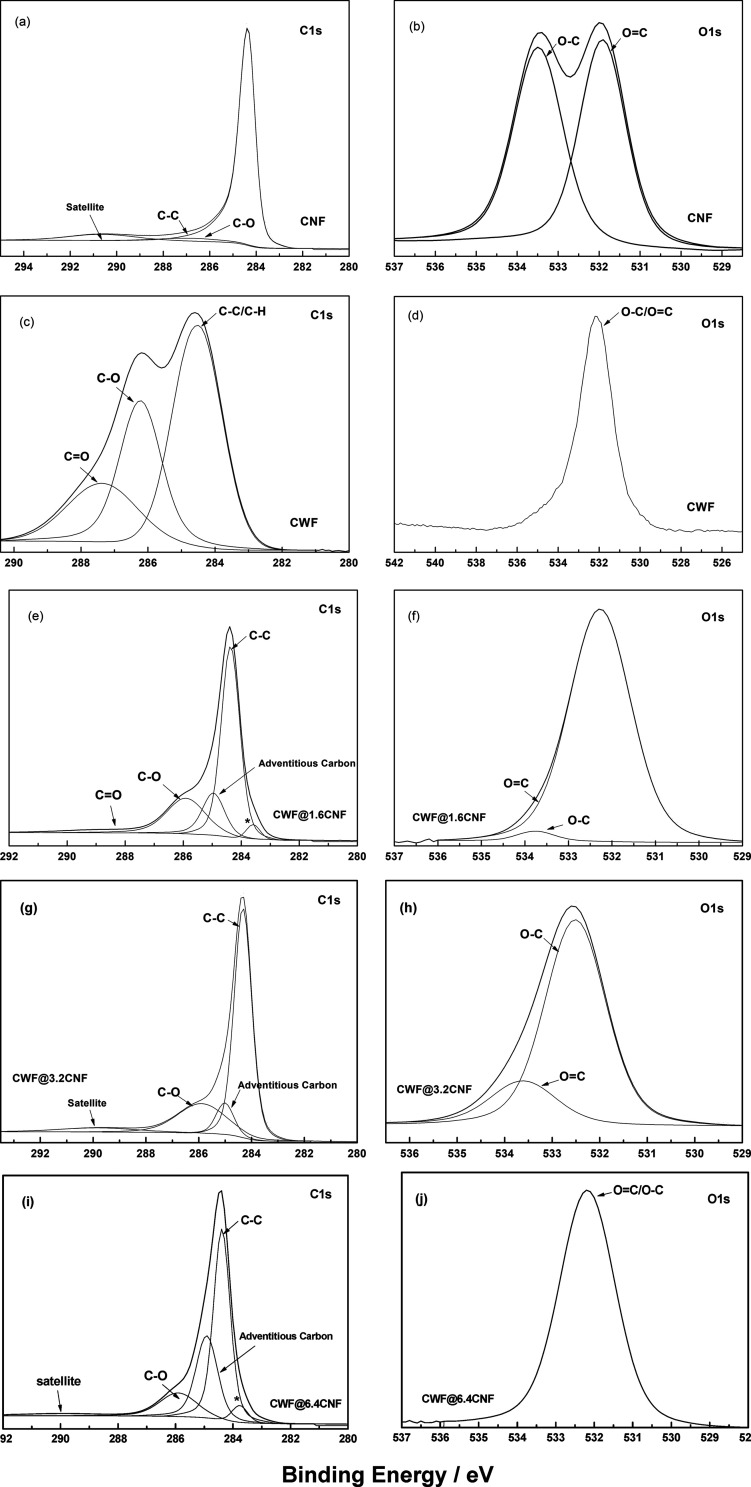
XPS deconvolution of CNFs, CWF, CWF@1.6 CNF, CWF@3.2 CNF, and CWF@6.4 CNF: (a) CNFs C 1s and (b) O 1s, (c) CWF C 1s and (d) O 1s, (e) CWF@1.6 CNF C 1s and (f) O 1s, (g) CWF@3.2 CNF C 1s and (h) O 1s, and (i) CWF@6.4 CNF C 1s and (j) O 1s.

**Figure 6:**
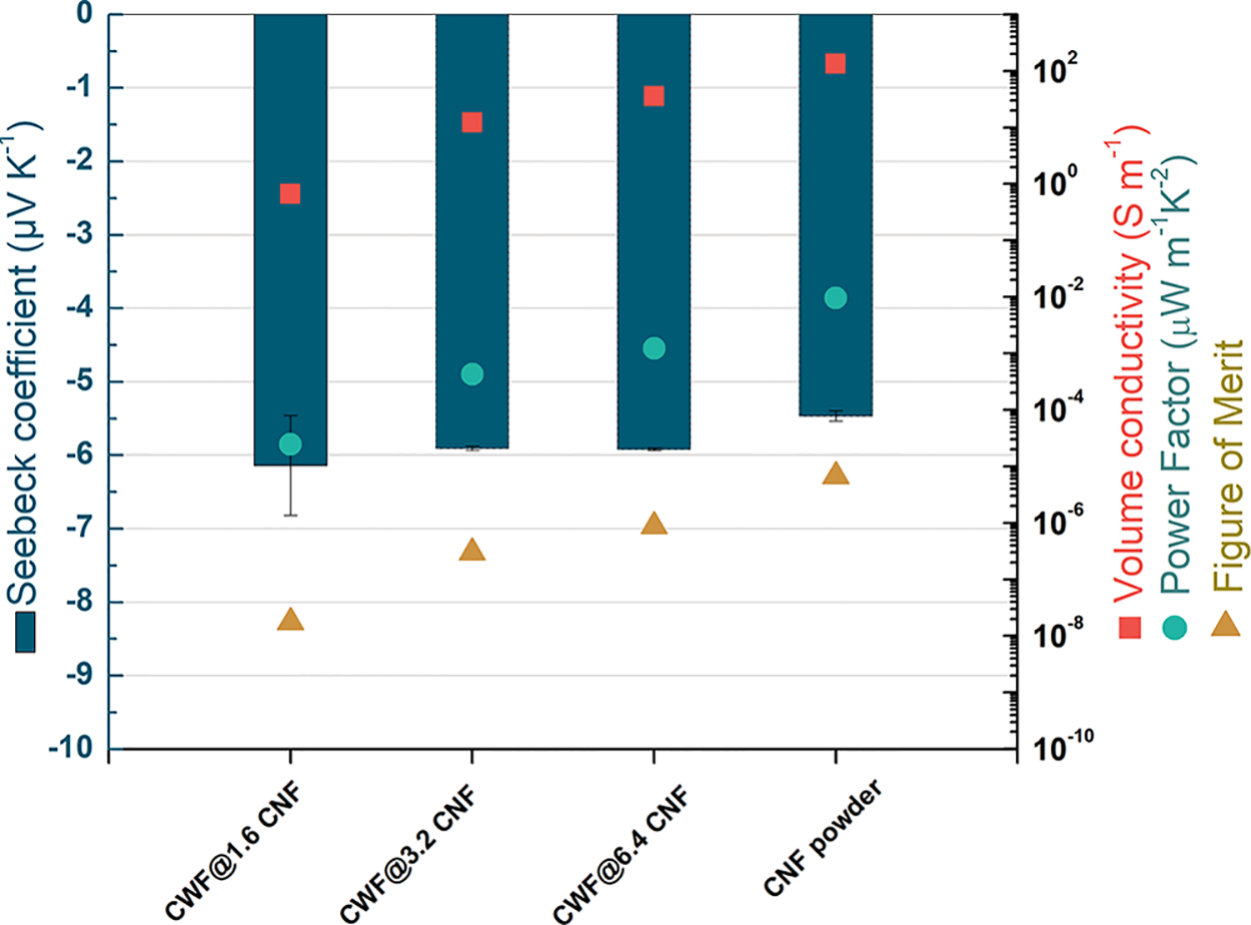
Electrical conductivity (squared symbols), Seebeck coefficient (rectangular bars), power factor (circle symbols), and figure of merit (triangle symbols) of e-textiles and CNF powder at 30 °C.

A comparison of the deconvolution of C 1s and O 1s spectra for as-received CNFs, CWF, and samples CWF@1.6 CNF, CWF@3.2 CNF, and CWF@6.4 CNF is presented in Figure 5. The C 1s spectra of the CNF showed a strong line at ∼284.4 eV (C—C), which, together with the “satellite” peaks, represents sp^2^ hybridized carbon (Figure 5a). An additional contribution from C—O (286.9 eV) was also observed.^24^ The C 1s spectra of the CWF reveal peaks at 284.5, 286.2, and 287.4 eV, attributed to (C—H), (C—O), and (O—C—O and/or C=O), respectively.^25^ As expected, the C 1s spectra of the CWF@1.6 CNF, CWF@3.2 CNF, and CWF@6.4 CNF samples present the signatures of the two base materials CWF and CNF. Though, two additional peaks at ∼285 eV, assigned to adventitious carbon, and ∼283.6 eV (labeled with * in Figure 5e,i), assigned to sp^2^ carbons, were also found in CWF@1.6 CNF and CWF@6.4 CNF. It is noteworthy that π–π* peaks are also detected in the C 1s spectra recorded for CWF@3.2 CNF and CWF@6.4 CNF (Figure 5g,i), which means that the signal from the CNFs is stronger in those samples. The O 1s spectra in as-received CNFs (Figure 5b) yielded peaks at ∼531.9 and ∼533.5 eV assigned to C=O and C—O, respectively,^26^ whereas in CWF, the peak at 532.1 eV can be associated with both C—O and C=O, which it is then shifted to ∼532.2 eV in CWF@6.4 CNF (Figure 6j).^27^ In summary, the Raman and XPS analysis seem to match well with the SEM images. Thus, the conductive ink used for producing the CWF@3.2 CNF samples may facilitate a deeper penetration of CNFs than the formulation used in CWF@6.4 CNF, where a larger amount of CNFs remain on the surface of the cotton woven fabrics.

### 3.3 Electronic Properties of e-Textiles at 30 °C

The electronic properties at 30 °C of the e-textiles and as-received CNF powder are represented in Figure 6 and Table 4. In terms of σ (presented as squared symbols in Figure 6), the CNF powder shows a σ = 133.5 ± 0.4 S m^–1^, correspondent to an electrical resistivity of ∼7.5 × 10^–1^ Ohm cm, which is 2 orders of magnitude higher than the value of 4 × 10^–3^ Ohm cm reported for individual Pyrograf III CNFs.^14^ It must be noticed that the setup used in this study only allows for evaluating the electrical conductivity of the as-received CNFs in their powder form. Thus, the values of σ reported here correspond to average values of CNF agglomerates. The σ found for the CNF powder is comparable to the electrical conductivity of some nitrogen-doped multiwall carbon nanotubes (MWCNTs), where values of ∼160 S m^–1^ were reported.^15^ The e-textiles showed σ from 0.70 ± 0.01 S m^–1^ corresponding to CWF@1.6 CNF samples to 35.4 ± 1.1 S m^–1^ of CWF@6.4 CNF samples. As expected (Table 4), the σ of the e-textiles is significantly lower than the σ of the CNF powder (133.5 S m^–1^). The presence of the insulating cotton fabrics and the discontinuities and imperfections of the coated CNF layer must hamper the appropriate creation of electronic pathways and thus explain the drop of σ observed for the conductive fabrics. The higher amount of CNFs (6.4 mg mL^–1^) used in the preparation of the conductive inks of CWF@6.4 CNF samples must be, on the other hand, the reason for their enhanced σ (with respect to the CWF@1.6 CNF and CWF@3.2 CNF samples). It must be noticed that a higher σ of 6 × 10^2^ S m^–1^ has been reported for cotton fabrics sprayed with conductive inks composed of 40 wt % of highly graphitic CNFs (Pyrograf-III PR 25 HHT XT).^28^ However, the amount of CNFs used in the production of the conductive inks was considerably lower in this study (approximately 6.3 wt % for aqueous inks of 6.4 mg mL^–1^).

**Table 4:** Electrical Conductivity σ, Seebeck Coefficient *S*, Power Factor PF, and Estimated Figure of Merit zT of e-Textiles and CNF Powder at 30 °C

sample	σ (S m^–1^)	*S* (μV K^–1^)	PF (μW m^–1^ K^–2^)	*zT*
CWF@1.6CNF	0.7 ± 0.01	–6.14 ± 0.7	2.5 ± 0.5 × 10^–5^	1.7 × 10^–8^
CWF@3.2CNF	12.3 ± 0.2	–5.9 ± 0.03	4.3 ± 0.03 × 10^–4^	3.0 × 10^–7^
CWF@6.4CNF	35.4 ± 1.1	–5.9 ± 0.02	1.2 ± 0.03 × 10^–3^	8.7 × 10^–7^
CNF powder	133.5 ± 0.4	–5.3 ± 0.08	3.7 ± 0.1 × 10^–3^	2.6 × 10^–6^

The Seebeck coefficient of all samples at 30 °C is also presented as rectangular bars in Figure 6 and Table 4. The intrinsic n-type of the CNF powder (−5.30 ± 0.08 μV K^–1^) is significant since most of as-produced CNTs are p-type conducting materials due to their oxygen doping with the environment.^29^ This finding means that air-stable n-type carbon nanofibers can be obtained at large-scale by conventional CVD.^30^ Among the limited works that report CNTs with negative Seebeck without using any sort of n-type doping strategy, it should be noted that free-standing MWCNT films,^31^ and MWCNT buckypapers,^32^ both grown by CVD, have shown Seebeck coefficients of around −6 μV K^–1^. The e-textiles showed negative Seebeck coefficients as well from −6.14 ± 0.70 μV K^–1^ for CWF@1.6 CNF to −5.9 μVK^–1^ for CWF@3.2 CNF and CWF@6.4 CNF samples. Therefore, the *S* of e-textiles is higher (in absolute value) than the *S* of the as-received CNF powder (−5.3 ± 0.1 μV K^–1^). This finding led to the hypothesis that the cotton fabric host, despite its insulating character, could have an active role on the final *S* obtained in the e-textiles. In this respect, it was theoretically demonstrated that a slight n-doping from cellulose to hexagonal graphene flakes can be induced when the basal adsorption between cellulose monomers and available graphitic planes of graphene is propitious.^12^ Therefore, the n-type doping of cellulose from the cotton fabric to the outer graphitic layers of CNFs seems to be possible, and it could explain the very slight increase of *S* found in the e-textiles. In addition, it cannot be omitted that some residuals of SDBS are remaining in the e-textiles. It has been reported that, when SDBS molecules homogeneously cover the surface of single wall carbon nanotubes (SWCNTs), this enhances the transfer of electrons from the sodium atoms to SWCNTs.^33^ Thus, the remaining residuals of SDBS on the surface of the CNFs could also explain the higher levels of *S* observed in the e-textiles. Notably, the *S* (absolute value) of the e-textiles coincides with the values of 6.4 ± 0.5 μV K^–1^ presented in the work previously mentioned, where highly graphitic CNFs based inks were sprayed onto cotton fabrics.^28^ However, in that study, the e-textiles were not n-type materials as in this work. The power factor (*S*^2^σ) at 30 °C was calculated, and the results are shown in Figure 6 (circle symbols) and Table 4. The CNF powder presented the highest PF of 3.7 × 10^–3^ μW m^–1^ K^–2^, followed by the CWF@6.4 CNF with a PF of 1.2× 10^–3^ μW m^–1^ K^–2^. These values are lower than the PF of 2.5 × 10^–2^ μW m^–1^ K^–2^ achieved in cotton fabrics sprayed with inks composed of aleuritic acid and Pyrograf-III CNFs.^28^ The highest figure of merit  of 2.64 × 10^–6^ at 30 °C for the CNF powder, followed by a *zT* of 8.7 × 10^–7^ for CWF@6.4 CNF were estimated from the experimental σ and *S* obtained in this study and the thermal conductivity *k* reported for buckypapers prepared with Pyrograf-III PR 25 CNFs (0.43 W m^–1^ K^–1^).^16^ Comparatively, the highest *zT* obtained for CWF@6.4 CNF samples is lower than the *zT* of 1.7 × 10^–5^ estimated for the conductive textiles produced with the higher concentrated inks based on Pyrograf-III CNFs,^28^ when considering the same thermal conductivity (0.43 W m^–1^ K^–1^).

### 3.4 Electronic Properties of e-Textiles from 30 to 100 °C

**Figure 7:**
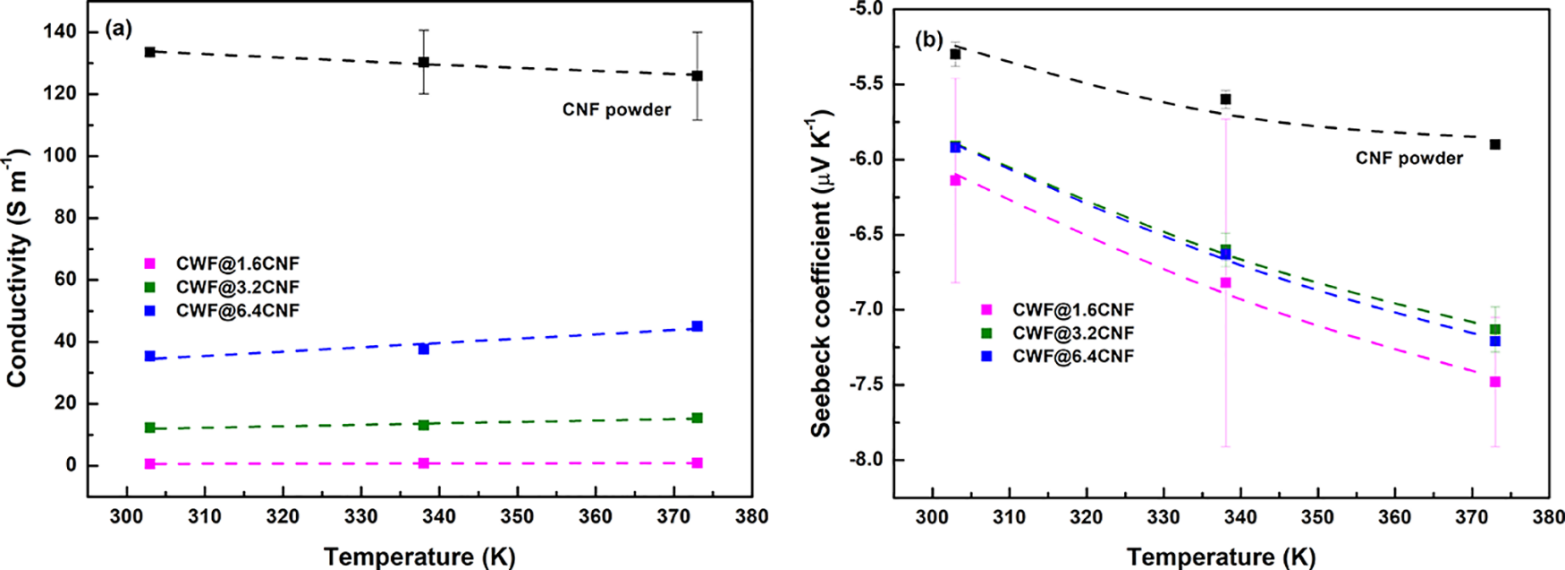
Electronic properties of CNF powder and e-textiles at temperatures from 303 to 373 K: (a) electrical conductivity and (b) Seebeck coefficient. The dash lines represent the fitting of σ(*T*) and *S*(*T*) with eqs 1 and 2, respectively.

The electronic properties (σ and *S*) of the e-textiles and as-received CNF powder from 303 K (30 °C) to 373 K (100 °C) are represented in Figure 7 to understand deeper their conduction mechanisms. As is shown in Table 4, a value of 133.5 ± 0.4 S m^–1^ wa obtained for the CNF powder at 30 °C (303.15 K), which decreases up to 125.9 ± 14.1 S m^–1^ at 100 °C (373.15 K). Interestingly, the CNF powder shows a positive temperature effect dσ/d*T* < 0 over the interval of temperatures. This is not expected since CNTs usually present dσ/d*T* > 0.^34−36^ In contrast, the e-textiles show a very slight increase in their conductivity with temperature (dσ/d*T* > 0). For instance, the σ(*T*) of the CWF@6.4 CNF increases from 35.4 ± 1.1 S m^–1^ at 30 °C (303.15 K) to 45.0 ± 0.4 S m^–1^ at 100 °C (373.15 K) (blue symbols in Figure 7a). Therefore, as shown in Figure 7a, a negative temperature effect effect applies for the case of e-textiles, which should make them useful as temperature sensors.^37^

The *S*(*T*) of the CNF powder is presented as square black symbols in Figure 7b. The n-type character of the CNF powder is found at all temperatures. In particular, the *S* of −5.3 μV K^–1^ observed at 30 °C increases gradually (in absolute value) up to −5.90 ± 0.03 μV K^–1^ at 100 °C. Moreover, the *S*(*T*) of the e-textiles, similarly to the *S*(*T*) of the CNF powder, shows a negative *S* that gradually is increasing (in absolute value) with temperature. Thus, the *S*(*T*) of the CWF@1.6 CNF increases from −6.14 μV K^–1^ at 30 °C to −7.5 ± 0.4 μV K^–1^ at 100 °C (purple symbols in Figure 7b). The larger standard deviation observed for the CWF@1.6 CNF samples can be explained by the lower homogeneity of their coated layers, when compared with the CWF@3.2 CNF and CWF@6.4 CNF samples.

### 3.5 Electronic Modeling of e-Textiles

The 3D variable range hopping (VRH) model is applied to evaluate the σ (*T*) nature of the CNF powder and e-textiles:^38,39^
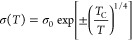
1Here, σ_0_ is the conductivity at an infinite temperature,  is a characteristic temperature scale determined by the average energy potential barrier (*W*_D_*< 0*) or potential well (*W*_D_ > 0), respectively, and *k*_B_ is the Boltzmann′s constant. It is important to notice that, when *W*_D_ > 0, eq 1 describes a thermally activated hopping mechanism across a random network of potential wells, leading to a typical dσ/d*T* > 0, while when *W*_D_ < 0, eq 1 describes a thermally activated scattering mechanism across a random distribution of impurities or structural defects, leading to a typical dσ/d*T* < 0. The corresponding values of σ_0_, *T*_C_, and *W*_D_ calculated from eq 1 are shown in Table 5 for all samples. Interestingly, the value of *T*_C_ (5.3 × 10^2^ K) is in the same order as the values reported for SWCNT mats (2.5 × 10^2^ K).^40^ Likewise, the *W*_D_ (absolute value) for the CNF powder (46 meV) is close to the activation energy (60 meV) reported for n-type graphitized carbon fibers in the 250–750 K interval.^41^ Notably, the CNF powder used in this study shows *W*_D_ < 0, which confirms the results found in a precedent work for CNFs Pyrograf III PR 19 LHT XT^26^ (it is reminded that the Pyrograf III PR 24 LHT XT grade is used in this study). This negative *W*_D_ can be explained by the presence of impurities such as the oxygen (∼1.8%) and sulfur (∼0.1%) detected by XPS. As it was previously discussed, these impurities could origin a thermal-enhanced backscattering mechanism due to the presence of virtual bound-states, represented as sharp peaks near the Fermi energy level *E*_F_ in the density of states.^42,43^ Likewise, the 3D VRH model has been used to evaluate the σ (*T*) of the e-textiles. Thus, as can be seen in Table 5, the *T*_C_ obtained for the e-textiles is 3 orders of magnitude higher than the *T*_C_ of the CNF powder (5.3 × 10^2^ K). Notably, the *W*_D_ of e-textiles is positive, in contrast to the negative *W*_D_ observed for the CNF powder. This fact implies that the σ(*T*) of e-textiles can be understood as the charge carriers overcoming the random network of potential wells by hopping.^39,44^ Therefore, it can be concluded that the σ(*T*) of e-textiles cannot be explained only in terms of the σ(*T*) found in CNF powder, but the cotton fabric or other factors (such as the remains of surfactant) must play its role in their mechanism conduction.

**Table 5:** Parameters σ_0_, *T*_C_, and *W*_D_ of CNF Powder and e-Textiles Obtained by Fitting the Experimental Values of σ(*T*) with the VRH Model [eq 1]

sample	σ_0_ (S m^–1^)	*T*_C_ (K)	*W*_D_ (eV)
CWF@1.6 CNF	635.6	6.8 × 10^5^	58.6
CWF@3.2 CNF	1353.3	1.5 × 10^5^	13.0
CWF@6.4 CNF	4730.3	1.8 × 10^5^	15.3
CNF powder	42.3	5.3 × 10^2^	–4.6 × 10^–2^

In this study, the *S*(*T*) of the CNF powder and e-textiles is depicted by the model proposed for describing the nonlinear Seebeck behavior of nitrogen-doped MWCNT mats:^42^
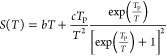
2where *bT* represents the metallic (linear) term of *S*(*T*), *c* is a constant, and *T*_P_ = (*E*_P_ – *E*_F_)/*k*_B_, where *E*_F_ is the Fermi energy level and *E*_P_ is the energy corresponding to the sharply varying and localized states near *E*_F_ in the density of states due to the contribution of impurities.^42,43^ The corresponding values of *b*, *c*, *T*_P_, and *E*_P_ – *E*_F_ calculated from eq 2 are shown in Table 6 for all samples. The best fit of *S*(*T*) for the CNF powder shows that the first term is positive with *b* = 5.6 × 10^–3^ μV K^–2^, while the second term is negative with *c* = −1.8 × 10^4^ μV and *T*_P_ = 988.2 *K*, yielding a *E*_P_ – *E*_F_ = 0.085 eV. It must be noticed that the negative sign of the constant *c* can be physically interpreted as the resonances near the *E*_F_ at the density of states caused by impurities present in the CNF structure.^42^ Likewise, as it is shown in Table 6, the parameters obtained with eq 2 for e-textiles are similar to the values calculated for the CNF powder. However, the e-textiles show a negative *b*, in contrast to the positive *b* of the CNF powder. Since eq 2 represents the contribution of two different transport mechanisms, where the positive sign of the parameter *b* corresponds to the charge of the nearly free (metallic) carriers, a n-type doping may be inferred in e-textiles, which must not be caused by the CNFs. As previously discussed in Section 3.3, this n-type doping may arise from the cellulose fibers of textile fabric or alternatively from the small amount of surfactant that still remains in the e-textile. It must be remarked that this latter assumption is based on the *b* > 0 observed in the CNF powder, where neither of these two potential donors (cellulose and surfactant) are present.

**Table 6:** Parameters *b*, *c*, *T*_P_, and *E*_P_ – *E*_F_ of CNF Powder and e-Textiles Obtained by Fitting the Experimental Values of *S*(*T*) with Equation 2

sample	*b* (μV K^–2^)	*c* (μV)	*T*_P_ (K)	*E*_P_ – *E*_F_ (eV)
CWF@1.6 CNF	–2.8 × 10^–3^	–1.7 × 10^4^	1077.1	9.3 × 10^–2^
CWF@3.2 CNF	–1.2 × 10^–3^	–1.7 × 10^4^	1061.1	9.1 × 10^–2^
CWF@6.4 CNF	–2.0 × 10^–3^	–1.7 × 10^4^	1075.1	9.3 × 10^–2^
CNF powder	5.6 × 10^–3^	–1.8 × 10^4^	988.2	8.5 × 10^–2^

## 4 Conclusions

In this study, the electrical conductivity (σ) and Seebeck coeficcient (*S*) between 30 and 100 °C of as-received carbon nanofiber (CNF) powder and therefrom derived e-textiles prepared by dip-coating with aqueous inks made with those CNFs and anionic surfactant were analyzed. At 30 °C, the σ, *S*, and power factor (PF) of the as-received CNFs are ∼133 S m^–1^, −5.3 μV K^–1^, and 3.7 × 10^–3^ μW m^–1^ K^–2^, respectively. The e-textiles prepared with a higher amount of CNFs (6.4 mg mL^–1^) show lower conductivities of 35 S m^–1^ but a higher *S* (absolute value) of −6 μV K^–1^, corresponding to a PF of 1.2 × 10^–3^ μW m^–1^ K^–2^ at 30 °C. Thus, not only the used CNF powder but also the e-textiles represent n-type materials with electrons as majority carriers. The origin of their n-type character is explained by the presence of some impurities found in the CNFs, which could produce sharp peaks close to the Fermi energy level (*E*_F_) in their density of states. Moreover, in contrast to the positive temperature effect found in the as-received CNFs, the σ(*T*) of the e-textiles from 30 to 100 °C shows a negative temperature effect. Therefore, it is deduced that the σ(*T*) of e-textiles cannot be explained only in terms of the σ(*T*) found in CNF powder, but the cotton fabric or other factors (such as the residuals of the used surfactant) must play their parts in their mechanism conduction. This finding is better understood through the 3D variable range hopping model of their σ(*T*), which points toward the charge carriers overcoming a random network of potential wells by hopping. In addition, the *S*(*T*) of the e-textiles from 30 to 100 °C presents a negative temperature effect, as is the case with the *S*(*T*) of the CNFs for the same range of temperatures. Moreover, it is deduced by applying the model proposed for describing the nonlinear Seebeck behavior of a certain sort of doped MWCNT mats that the e-textiles may have a n-type doping arising from the cellulose fibers of the textile fabric or from the remaining residuals of surfactant used in the formulation of the aqueous inks.
